# Combination therapy with ultrasound and 2D nanomaterials promotes recovery after spinal cord injury via *Piezo1* downregulation

**DOI:** 10.1186/s12951-023-01853-y

**Published:** 2023-03-15

**Authors:** Feng Zhang, Xiaolie He, Kun Dong, Li Yang, Bei Ma, Yuchen Liu, Zhibo Liu, Bairu Chen, Rongrong Zhu, Liming Cheng

**Affiliations:** 1 Department of Orthopaedics, School of Medicine, Tongji Hospital Affiliated to Tongji University, Tongji University, Shanghai, 200065 China; 2 Key Laboratory of Spine and Spinal Cord Injury Repair and Regeneration of Ministry of Education, School of Life Sciences and Technology, Tongji University, Shanghai, 200065 China; 3 Frontier Science Center for Stem Cell Research, Tongji University, Shanghai, 200065 China

**Keywords:** Ultrasound, Nanoparticles, Spinal cord injury, *Piezo1*, Inflammation

## Abstract

Spinal cord injury (SCI) causes severe neurological dysfunction and currently has no effective treatment. Due to the complex pathophysiological processes associated with SCI and the limited efficacy of single strategies, the need for combined strategies for effective SCI therapy is becoming increasingly apparent. In this study, we evaluated the combined effects of layered double hydroxide-coupled NT3 (MgFe-LDH/NT3) nanoparticles (NPs) and ultrasound (US) both in vitro and in vivo. Combined treatment promoted neural stem cell (NSC) differentiation into neurons and exerted anti-inflammatory effects in vitro. Furthermore, combined therapy promoted behavioural and electrophysiological performance at eight weeks in a completely transected murine thoracic SCI model. Additional RNA sequencing revealed that ultrasonic-induced *Piezo1* downregulation is the core mechanism by which combined therapy promotes neurogenesis and inhibits inflammation, and the Piezo1/NF-κB pathways were identified. Hence, the findings of this study demonstrated that the combination of ultrasound and functional NPs may be a promising novel strategy for repairing SCI.

## Introduction

Spinal cord injury (SCI) is a serious nervous system disorder that causes a loss of sensory and motor function [1]. A hostile inflammatory environment forms following SCI, and the ensuing primary and secondary neuronal death, as well as the lack of neurotrophic factors, complicates the ability of the spinal cord to repair itself [2, 3]. Presently, commonly used clinical practices, such as neurotrophic drugs, surgery, and rehabilitation exercise, have unsatisfactory effects [4]. Therefore, the treatment of SCI remains challenging.

Recently, the development of nanomaterials has brought new hope to patients with SCI. Nanomaterials induce nerve axon regeneration and inhibit the formation of glial scars by carrying stem cells, neurotrophic factors, and drugs, achieving curative effects [5, 6]. Layered double hydroxides (LDHs), which are 2D nanomaterials, have recently attracted attention due to their remarkable bioactive activities [7]. LDH presents substantial biological properties, such as good biocompatibility, safe biodegradation, and high drug-loading efficiency. The LDH-based delivery system can protect vulnerable factors from degradation, in turn improving bioavailability [8, 9]. According to our previous studies [10], MgAl-LDH/NT3 implantation significantly improves the behavioural and electrophysiological performance of mice with SCI. New neurons were also found in the lesion sites; hence, MgAl-LDH/NT3 presents notable potential for treating SCI.

Physical therapies, such as ultrasound (US), have also attracted attention for SCI treatment. US is a noninvasive technology commonly used in clinical applications. Increasing evidence demonstrates that US might serve as a potential anti-inflammatory technique in many diseases [11]. According to a previous study, the mechanical stimulus applied by low-intensity pulsed US exerts an anti-inflammatory effect on lipopolysaccharide (LPS)-treated osteoblasts [12]. Additionally, US can alleviate proinflammatory factor expression induced by LPS in macrophages via the TLR4/NF-κB pathway [13]. The inflammatory environment at the lesion site plays a detrimental role in SCI, as it accelerates neuronal apoptosis and demyelination of axons and hinders neurogenesis [14, 15]. However, to the best of our knowledge, little research regarding the application of US for SCI treatment exists due to its limited effects on nerve regeneration.

**Scheme 1:**
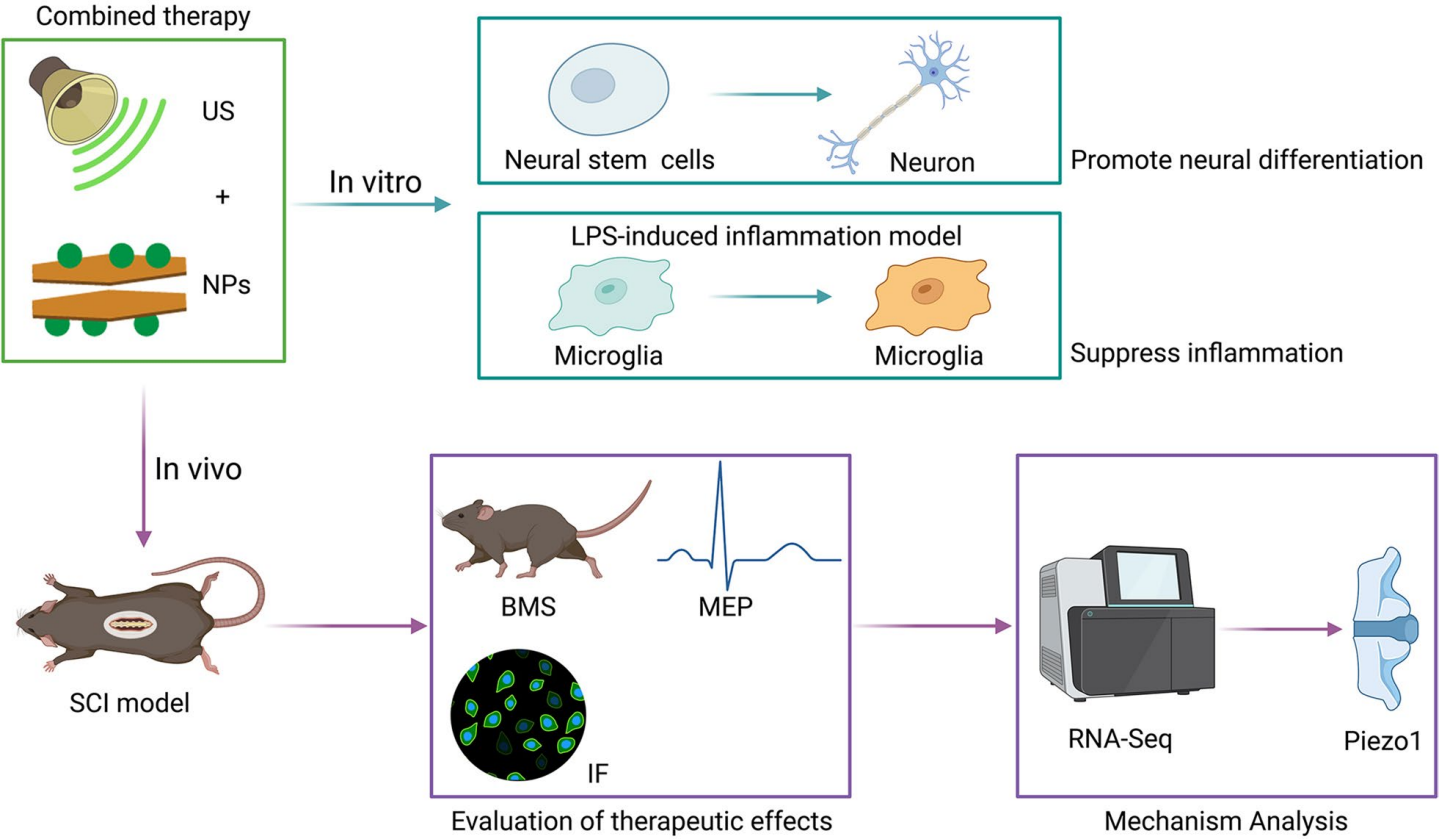
Sequential scheme of the studies performed. MgFe-LDH/NT3 nanoparticles (NPs) were synthesized and then combined with ultrasound (US), and the effects of combined therapy were tested both in vitro and in vivo. In vitro, the roles of promoting neural differentiation and suppressing inflammation were investigated. In vivo, the therapeutic effects of combined therapy on SCI model mice were analyzed, further Piezo1 was identified via RNA-seq analysis and the underlying mechanisms were revealed then. The figure was created with BioRender.com

The complex microenvironment at the lesion site following SCI is not ideal for the recovery of neurological function, although a single approach has achieved some effects. Increasing evidence suggests that combined strategies are superior to single strategies [16, 17]. Combining biomaterials and physical procedures provides greater benefits in SCI treatment than either procedure applied alone [18]. Combined therapy using multiple treatments simultaneously may be required to achieve satisfactory therapeutic effects. In this study, we used a modified LDH (MgFe-LDH) [19] loaded with neurotrophic factor 3 (NT3) to obtain MgFe-LDH/NT3. MgFe-LDH/NT3 was further transplanted into the injury site of an SCI murine model and US treatment was then performed to better promote functional recovery and neurogenesis. We also explored the biological effects of the combined treatment on NSC differentiation, inflammation regulation, and the potential therapeutic mechanism (Scheme 1).

## Results

### Characterization of MgFe-LDH/NT3

**Fig. 1:**
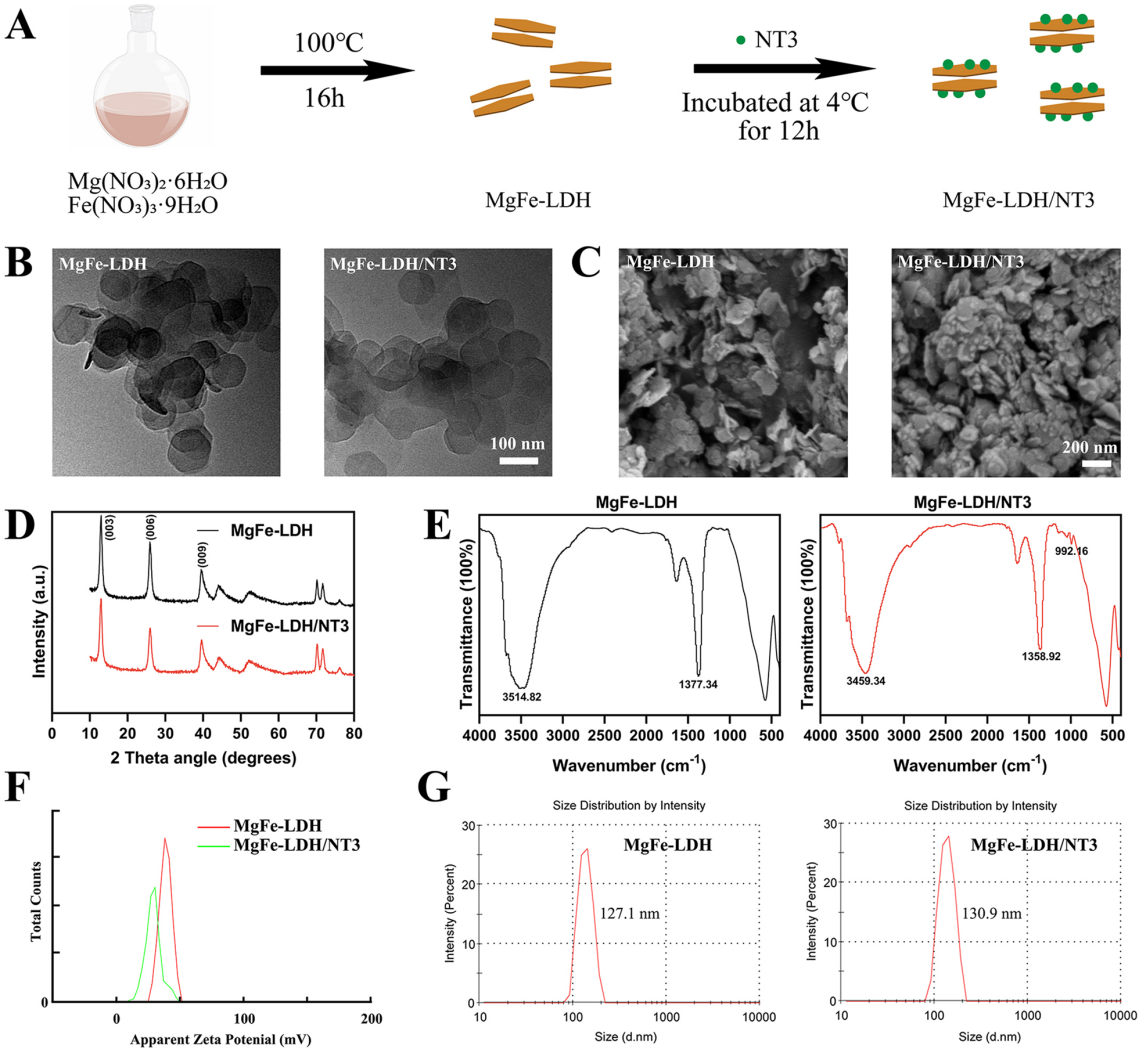
Preparation and characterization of nanoparticles. **A** Schematic illustration of MgFe-LDH/NT3 preparation. **B** TEM images of MgFe-LDH and MgFe-LDH/NT3. **C** SEM images of MgFe-LDH and MgFe-LDH/NT3. **D** XRD patterns of MgFe-LDH and MgFe-LDH/NT3. **E** FTIR spectra of MgFe-LDH and MgFe-LDH/NT3. **F** Zeta potential distribution of MgFe-LDH and MgFe-LDH/NT3.** G** Particle size distribution of nanoparticles

A schematic illustration of the MgFe-LDH/NT3 nanoparticles (NPs) preparation is shown in Fig. 1A and is described in detail in the methods section. The synthesized NPs were then serially characterized. As shown in the transmission electron microscopy (TEM) images (Fig. 1B), both MgFe-LDH and MgFe-LDH/NT3 presented a layered hexagonal morphology. Furthermore, the scanning electron microscopy (SEM) images (Fig. 1C) demonstrate the surface morphology of MgFe-LDH and MgFe-LDH/NT3, which were also layered hexagonally, consistent with the TEM images. The X-ray diffraction (XRD) analysis (Fig. 1D) revealed characteristic peaks of (003), (006), and (009) in both MgFe-LDH and MgFe-LDH/NT3, indicating successful construction of the layered structure. As shown in the Fourier transform infrared (FTIR) spectrum (Fig. 1E) for MgFe-LDH and MgFe-LDH/NT3, the stretching vibration peaks of –OH occurred at 3514.82 cm^−1^ and 3459.34 cm^−1^, respectively. Furthermore, the stretching vibration peaks of –NO_2_ were at 1377.34 cm^−1^ and 1358.92 cm^−1^. One notable peaks of C–H occurred at 992.16 cm^−1^ in MgFe-LDH/NT3 only, which means that organic compounds such as proteins were introduced into the synthetized materials, confirming successful loading of NT3. According to the results of zeta potential detection (Fig. 1F), the average zeta potentials of MgFe-LDH and MgFe-LDH/NT3 were 38.1 mV and 29.2 mV, respectively, which was largely due to the negative potential of NT3. The average diameters of the MgFe-LDH and MgFe-LDH/NT3 nanoparticles were 127.1 and 130.9 nm (Fig. 1G), respectively. Loading efficiency of NT3 in MgFe-LDH is 99.775% using a ELISA Kit for NT3. Taken together, these results indicated that our synthetization was successful.

### Combined treatment enhanced the directed differentiation of NSCs into neurons

**Fig. 2:**
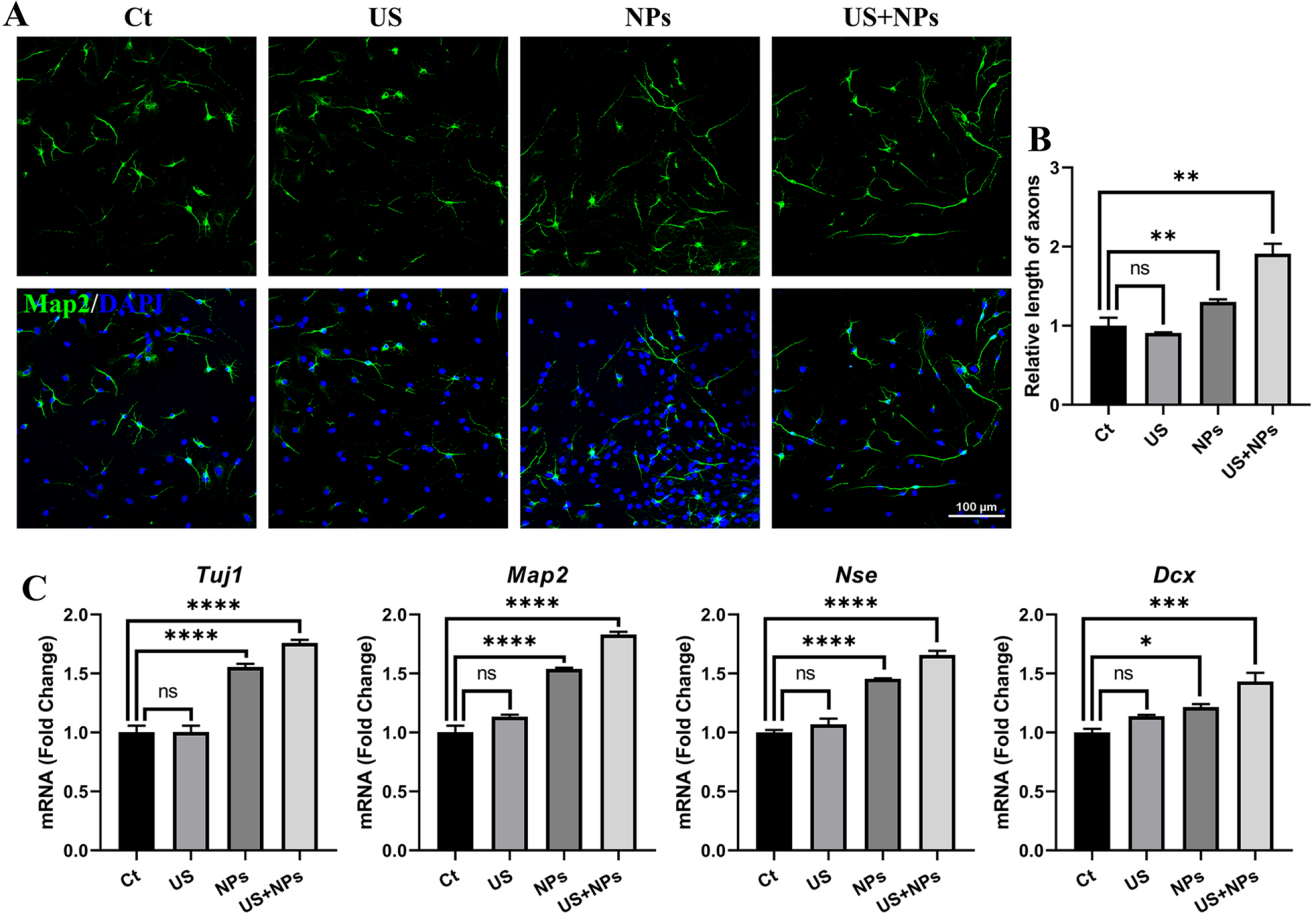
Combined treatment promoted directed differentiation of NSCs into neurons in vitro. **A** Immunofluorescence images of Map2-positive (green) cells in spontaneous differentiation medium. Cell nuclei were stained with DAPI (blue). **B** Quantification of the relative length of axons as compared to the Ct group. **C** The mRNA expression of neuronal markers (*Tuj1*, *Map2*, *Nse*, *Dcx*) was analysed by qPCR. Ct, control; US, ultrasound; NPs, nanoparticles

According to one of our previous studies, MgAl-LDH and MgAl-LDH/NT3 can promote neurogenesis in mice with SCI [10]; in another of our studies, MgFe-LDH NPs were superior to MgAl-LDH because the requirements and tolerance of aluminium are far below those of iron [19]. Thus, MgFe-LDH was employed in the present study. To investigate the effects of the combined treatment containing MgFe-LDH/NT3 NPs and US on neural differentiation, neural stem cells (NSCs) were incubated with differentiation medium continuously for seven days and exposed to different treatments. Map2 was used as a neuronal marker, and as shown in the immunofluorescence staining results (Fig. 2A, B), compared with the Ct group, the length of axons in the US-treated group (US) showed no obvious extension. The MgFe-LDH/NT3 NP-treated group exhibited longer axons. The combined treatment in the US + NPs group promoted axon extension, which indicated that the combined treatment played a better role in promoting NSC differentiation than the treatments used alone. In addition, the mRNA expression levels (Fig. 2C) and qPCR results were consistent with the immunofluorescence staining results. Compared with the Ct group, US alone did not alter the gene expression significantly. Both NPs and combined treatment promoted the gene expression of the neuronal markers *Tuj-1*, *Nse*, *Dcx,* and *Map2*, as well as elevated levels of gene expression. These results indicate that combined treatment can accelerate the neural differentiation of NSCs, which may benefit neural disease treatment.

### Combined treatment decreased proinflammatory cytokines in LPS-induced SIM-A9 cells

**Fig. 3:**
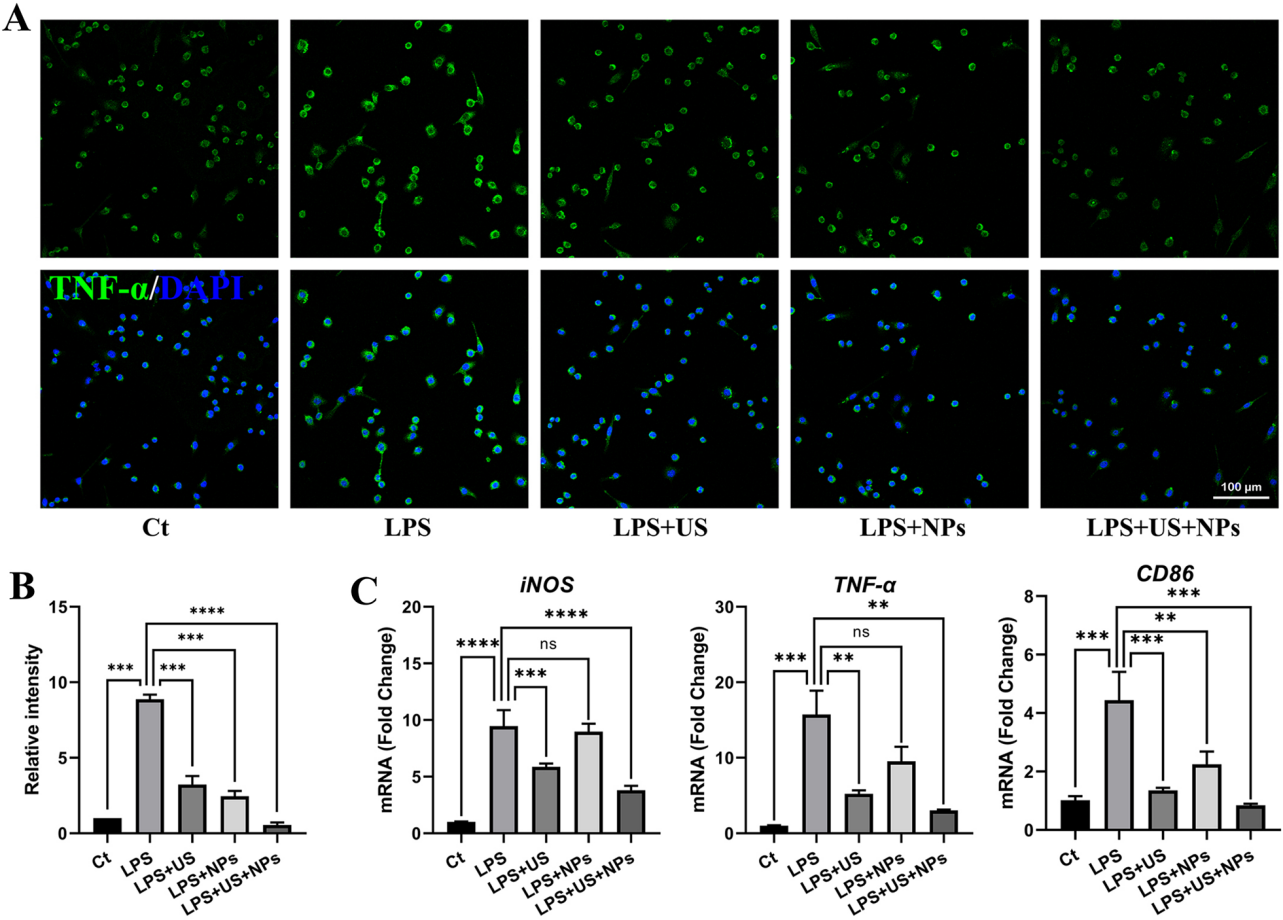
Combined treatment suppressed inflammation induced by LPS in SIM-A9 cells in vitro. **A** Confocal laser scanning microscopy images for immunofluorescence staining of SIM-A9 cells treated with LPS, LPS + US, LPS + NPs, and LPS + US + NPs. Cell nuclei were stained with DAPI (blue), and TNF-α was stained with a green signal. **B** Quantification of TNF-α relative fluorescence intensity in each group. **C** The expression of inflammation markers (*iNOS*, *TNF-α*, and *CD86*) was analysed by qPCR

Our previous studies have also indicated that MgAl-LDH/NT3 can inhibit inflammatory responses in mice with SCI [10]. Here, we investigated whether the combined treatment would confer synergistic effects because some reports indicate that US can also suppress inflammatory responses [20, 21]. To assess the anti-inflammatory effect of the combined treatment, LPS was used to establish a microglial SIM-A9 cell inflammation model, and immunofluorescence staining of proinflammatory cytokines was conducted. As shown (Fig. 3A, B), the fluorescence intensity of tumour necrosis factor alpha (TNF-α) in the LPS group was stronger than that in the control group, indicating that LPS successfully induced inflammation in SIM-A9 cells. Compared to that in the LPS group, the fluorescence intensity in the NPs, US, and US + NPs groups was significantly lower, and the intensity in the US + NPs group was the lowest, even lower than that in the Ct group. Furthermore, *TNF-α, iNOS,* and *CD86* mRNA expression was analysed using qPCR (Fig. 3C). Compared to the Ct group, LPS induced *TNF-α, iNOS,* and *CD86* upregulation*.* In contrast, US significantly decreased the expression levels. NPs, however, were not as effective as US in decreasing the expression level, and combined treatment exerted better anti-inflammatory activities than US or NPs alone, which indicated the effective anti-inflammatory ability of combined treatment.

### Combined therapy promoted functional recovery in mice after SCI

**Fig. 4:**
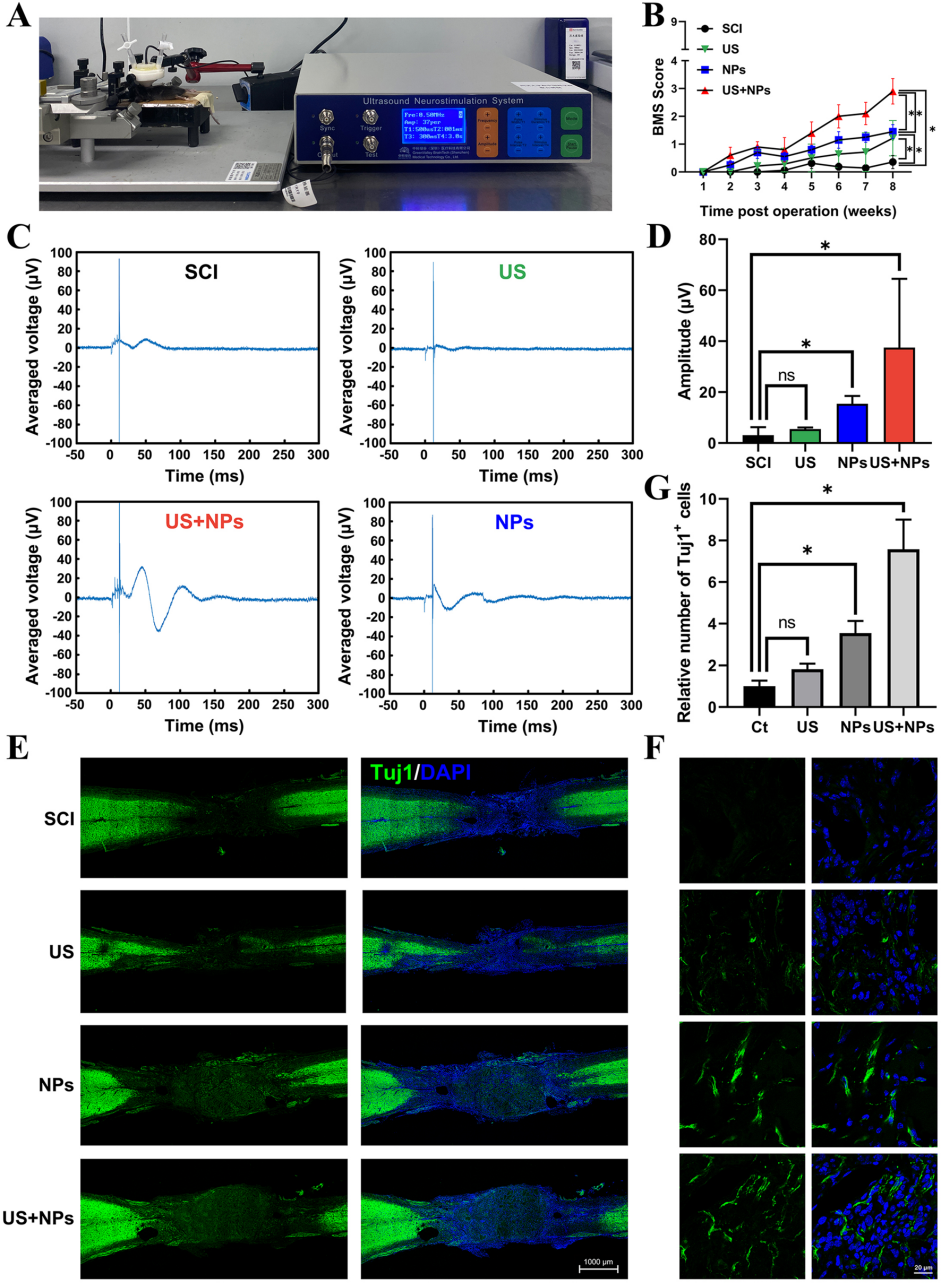
Combined therapy promoted the recovery of motor function and neurogenesis in SCI mice. **A** Mice with SCI and NPs transplantation exposed to ultrasound, namely, the US + NPs group. **B** Mouse motor functional recovery using BMS scores for statistical analysis.** C** Electrophysiological analysis of the mice with different treatments after SCI. **D** Amplitude of MEPs. **E** Representative immunofluorescence staining of spinal cord sections eight weeks after surgery. Cell nuclei were stained with DAPI (blue). Tuj1 (green) was used as a neuron marker. **F** Amplified images of the lesion site in each group.** G** Quantification of Tuj1 positive neural cells in lesion site

The above findings suggested that the combined treatment could better promote neural differentiation and exert an anti-inflammatory effect in vitro. To evaluate the in vivo function, SCI models were subjected to combined therapy (Fig. 4A), where the mice with SCI transplanted with NPs received US stimulation. The motor functional recovery of SCI mice was evaluated with the Basso Mouse Scale (BMS) (Fig. 4B). At eight weeks, US, NPs, and combined therapy significantly improved functional recovery compared with that in the SCI group. Recovery was evident in the US + NPs group, with a BMS score of 2.75, which represented the best functional recovery among all groups. Next, as shown in Fig. 4C, D, an electrophysiological assay was carried out to analyse neural conduction by stimulating the brain and recording hind limb motor evoked potential (MEP). The average amplitudes of MEP were 3.1, 5.5, 15.4, and 37.4 μV for the SCI group, US group, NPs group, and US + NPs group, respectively. Combined treatment thus could better promote neural conduction activities in mice with SCI. In addition, we explored whether combined therapy could promote neurogenesis following SCI. Tuj1, a mature neuron marker, was stained to investigate neurogenesis at the lesion site. As shown in Fig. 4E–G, Tuj1 signals were observed in the NPs and US + NPs groups, which indicated neurogenesis. Combined, these results suggest that combined therapy can improve motor functional recovery and promote neurogenesis.

### RNA sequencing revealed the downregulation of Piezo1 after combined therapy

**Fig. 5:**
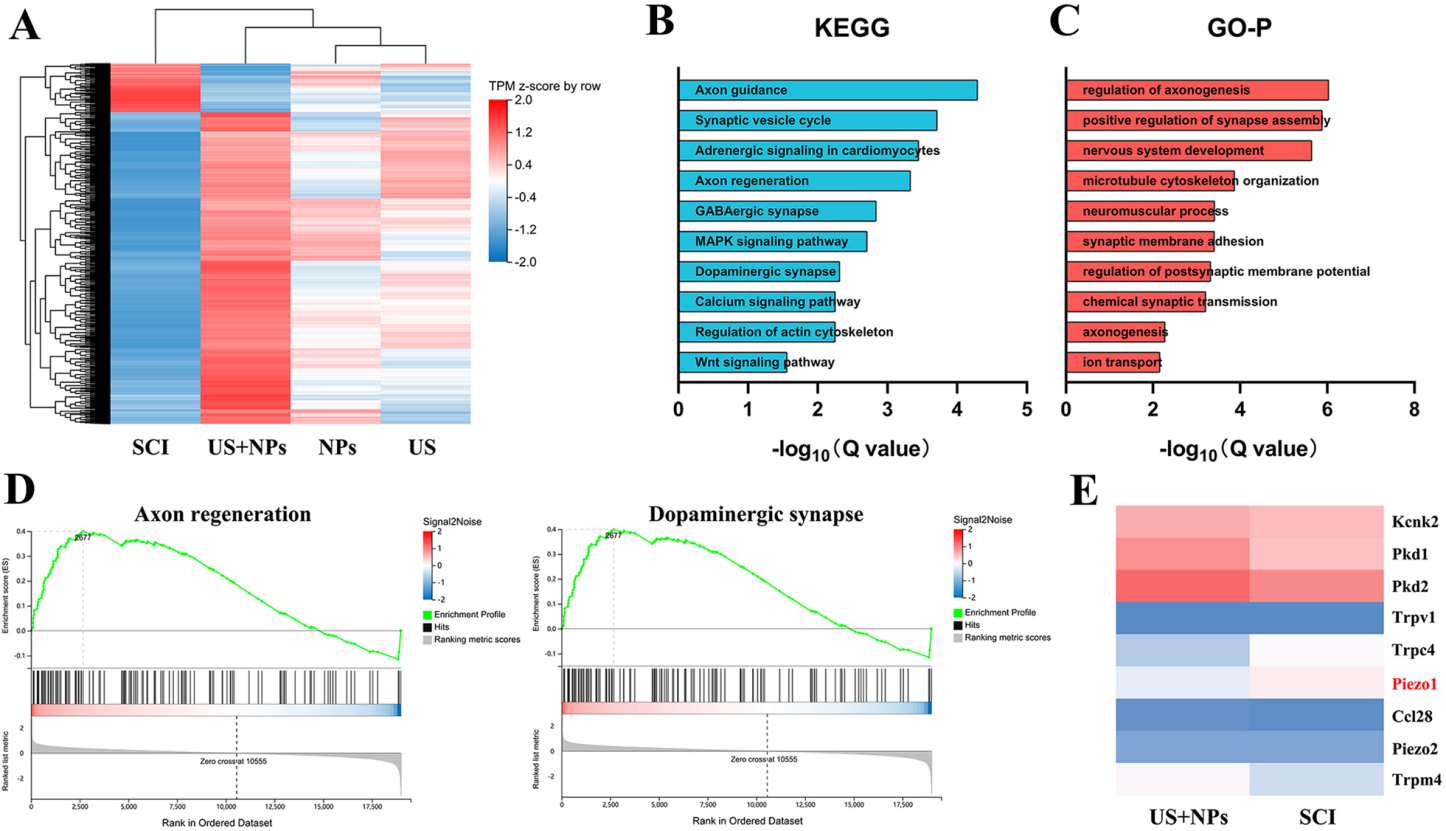
RNA-seq analysis of US + NPs treated mice with SCI. **A** Hierarchical clustering heatmap of DEGs. **B** KEGG pathway enrichment analyses. **C** GO enrichment analyses. **D** GSEA between the US + NPs group and SCI group. **E** Heatmap of ultrasound-related genes in the US + NPs group and SCI group

Although we proved that combined therapy could promote motor functional recovery, the associated mechanism still requires further explanation. Consequently, we performed RNA-seq to determine important genes or pathways involved by analysing the differentially expressed genes (DEGs). According to the heatmap from Fig. 5A, a notable distinction in expression patterns was found upon comparing the SCI and US + NPs groups. Furthermore, the DEGs between the SCI and US + NPs groups were selected for sequential analysis. KEGG pathway analysis revealed that DEGs were mostly enriched in axon guidance and axon regeneration calcium signalling pathways, among others (Fig. 5B). The GO-BP terms were related to regulation of axonogenesis, positive regulation of synapse assembly, and nervous system development (Fig. 5C). Additionally, GSEA results indicated a positive normalized enrichment score (NES) for gene sets associated with neural repair, such as axon regeneration and dopaminergic synapses, suggesting that combined therapy enhanced neural regeneration in mice with SCI (Fig. 5D). Furthermore, we evaluated US-related gene expression in the SCI and US + NPs groups, and a heatmap is presented in Fig. 5E. *Piezo1* was identified as a DEG between the SCI and US + NPs groups. *Piezo1* has been reported to be closely related to neurogenesis [22, 23].

### Combined therapy downregulated Piezo1 expression at the injury site in mice after SCI

**Fig. 6:**
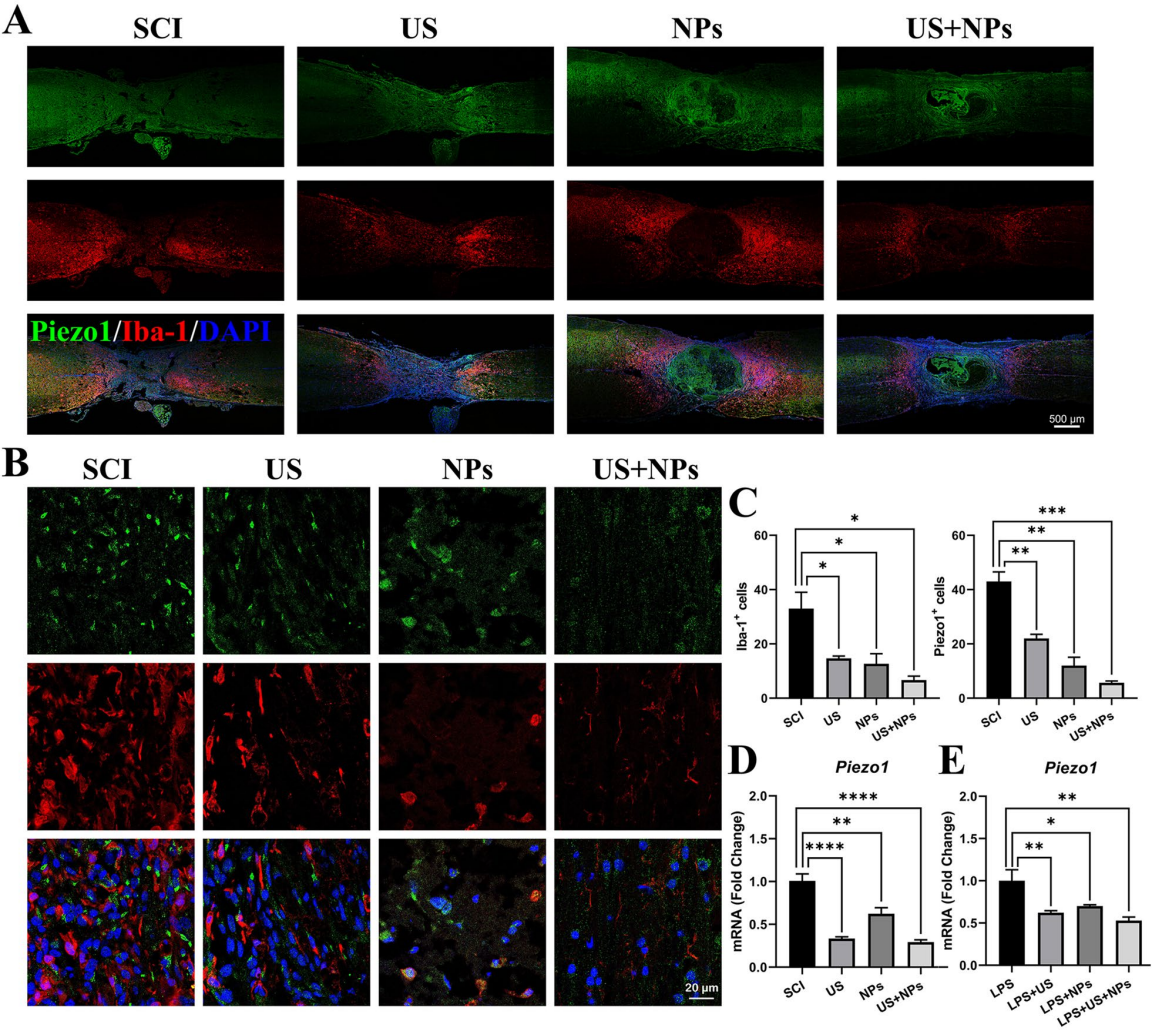
Combined therapy decreased the expression of *Piezo1* at the lesion site. **A** Immunostaining of Piezo1 (green) and Iba1 (red) in the mouse spinal cord. Cell nuclei were stained with DAPI (blue). **B** Amplified images of regions at the lesion site of mice. **C** Quantification of Iba1^+^ and Piezo1^+^ cells in the lesion site for each group. **D** qPCR validation of *piezo1* expression in the lesion sites of various injured spinal cords in each group.** E** The expression of *piezo1* in LPS treated SIM-A9 cells in vitro was analysed by qPCR

According to the transcriptome sequence analysis, *Piezo1* was obtained. We verified *Piezo1* expression at the lesion sites using immunofluorescence and qPCR. Since *Piezo1* can mediate inflammation regulation [24, 25], we contained Piezo1 and the microglial marker Iba-1 to detect the inflammatory response (Fig. 6A, B) and determined the number of Iba-1-positive and Piezo1-positive cells at the lesion site (Fig. 6C). Compared with that in the SCI group, the numbers of Iba-1-positive and Piezo1-positive cells in the US, NPs, and US + NPs groups were significantly decreased. Combined therapy showed the highest effectivity in reducing the number. qPCR was then performed to investigate *Piezo1* gene expression at the lesion site. Compared with the SCI group, the NPs, US, and combined therapy groups all exhibited decreased *Piezo1* expression (Fig. 6D). We further determined *Piezo1* expression in LPS-induced SIM-A9 cells, and the results shown that combined treatment significantly decreased the expression of *Piezo1*, compared to the LPS group (Fig. 6E), which also support the in vivo data. The above results indicated that *Piezo1* was a possible key target for combined therapy, and this phenomenon may be largely related to the immunomodulatory capacity.

### Combined therapy alleviates inflammation in the lesion site after SCI via Piezo1/NF-κB

**Fig. 7:**
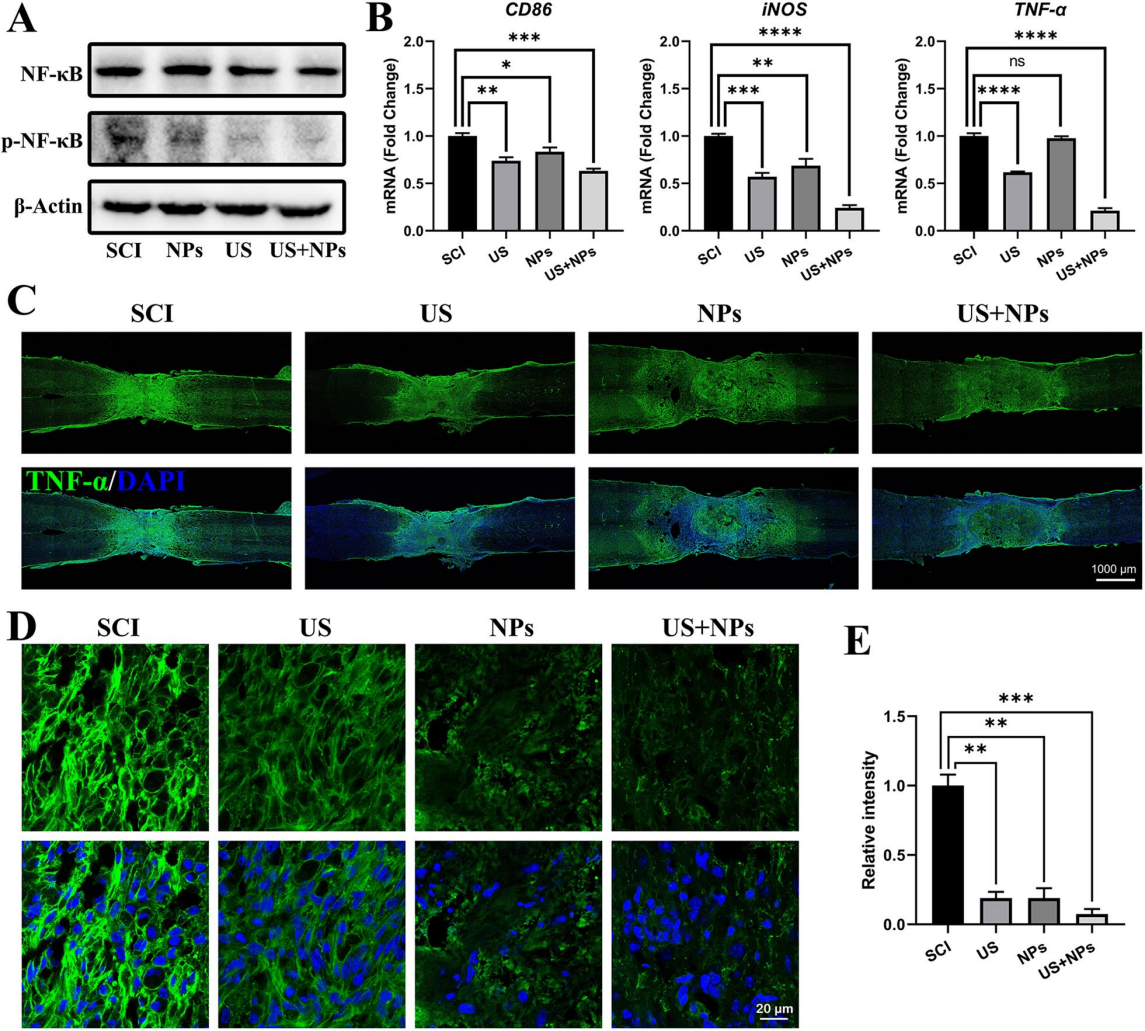
Combined therapy reduced inflammation at the injury site via the Piezo1/NF-κB pathways.** A** WB was used to assess NF-κB/pNF-κB protein levels. **B** The expression of inflammation markers (*CD86*, iNOS, and TNF-α) at the lesion site was analysed by qPCR one week after injury. **C** Representative immunofluorescence staining images for TNF-α (green) seven days after surgery. Cell nuclei were stained with DAPI (blue). **D** Amplified images of each group. **E** Relative intensity of TNF-α in the lesion site

Inflammation plays a key role during the process of SCI and can also be enhanced by Piezo1 activity. To explore the downstream pathway regulated by *Piezo1*, we determined NF-κB because it is an essential transcription factor in inflammatory and healing activation. Hence, western blotting was conducted to determine the protein expression levels of NF-κB and p-NF-κB at the lesion sites [24]. Excitingly, compared with the SCI group, the NPs, US, and US + NPs groups exhibited p-NF-κB downregulation (Fig. 7A), and p-NF-κB expression in combined therapy-treated mice was the lowest, indicating the notable anti-inflammatory effects of combined therapy. Next, we detected inflammation-related gene expression levels in lesion sites by qPCR. NPs significantly decreased *CD86* and *iNOS* expression, and the expression of *CD86*, *iNOS*, and *TNF-α* in the US and US + NPs groups was significantly downregulated compared with that in the SCI group (Fig. 7B). Finally, the expression of TNF-α at the lesion site was validated by immunofluorescence staining (Fig. 7C–E). Compared to SCI, a significant decrease in fluorescence intensity was observed in all groups, among which the function of the combined therapy was most notable. However, TNF-α expression were not in consistent in mRNA and protein level especially in NPs group, we speculate it mainly because of that they are in different dimensions. As we all know, multiple factors including post-transcriptional processing modifications of mRNA would affect the protein expression. To sum up, these results suggested that the combined therapy also had a certain anti-inflammatory effect in vivo and that this effect was enabled by the *Piezo1*-mediated NF-κB pathway.

### Biosafety assessment

**Fig. 8:**
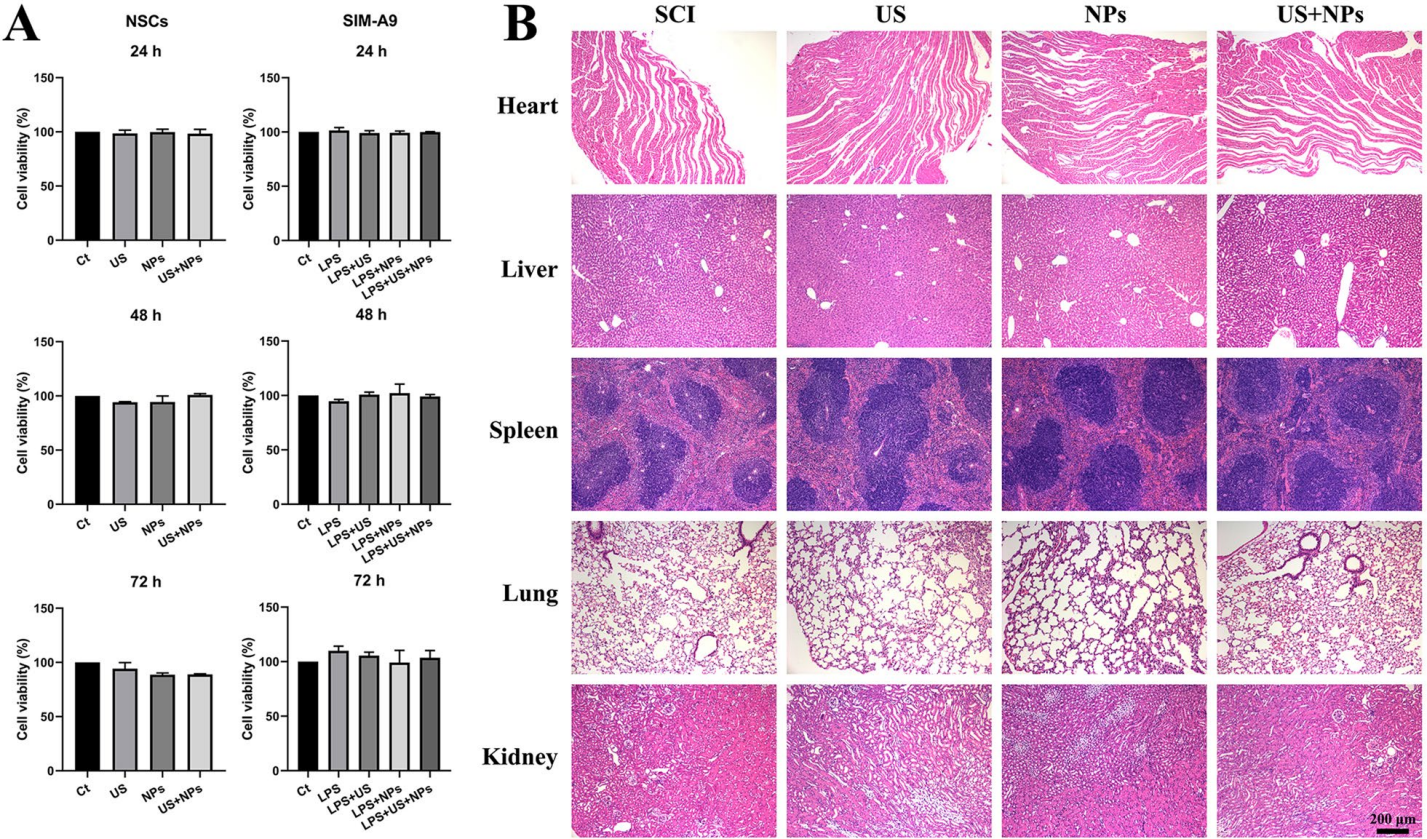
Biosafety assessment of combined therapy in vitro and in vivo. **A** Cell viability detected by CCK-8. **B** H&E staining of the tissue in all groups

Cell counting kit-8 (CCK-8) and histological examination were performed to explore the biosafety of the combined therapy in vitro and in vivo, respectively. The survival rate of NSCs and SIM-A9 treated for 24, 48, and 72 h was detected using a CCK-8 assay (Fig. 8A). No significant differences in cell viability were observed between these treatments both in NSCs and SIM-A9, showing that combined therapy would not cause any cytotoxicity. As shown in Fig. 8B, the morphology of tissues in the SCI, US, NPs, and US + NPs groups indicated no significant difference, demonstrating that our treatments were safe and may be applied in clinical settings in the future.

## Discussion

US has gained attention in many fields due to its noninvasiveness and minimal adverse effects. Several studies have revealed the potential of US for alleviating the inflammatory response [26, 27]; however, no obvious effects on treating SCI have been reported. Recently, biomaterials have also demonstrated therapeutic potential for application in SCI [10, 28–30], and MgAl-LDH/NT3 could promote mice function recovery from SCI in our previous study. To achieve better functional recovery, in this study, we implemented a novel strategy combining US and biomaterials for the treatment of SCI with satisfactory outcomes. US suppressed the inflammatory response both in vitro and in vivo, and MgFe-LDH/NT3 NPs promoted neurogenesis. Further analysis revealed that *Piezo1* is the core factor by which combined therapy promotes neurogenesis and inhibits inflammation.

**Fig. 9:**
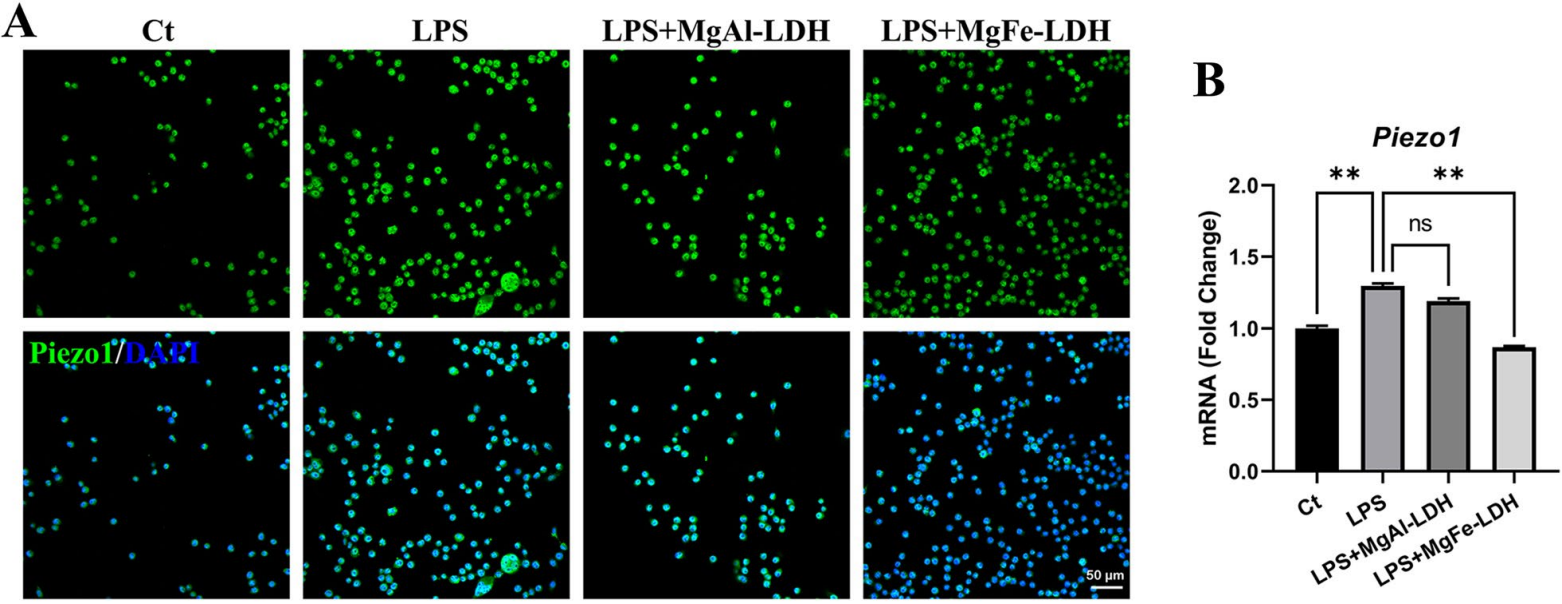
Comparison of Piezo1 expression in MgFe-LDH and MgAl-LDH treated SIM-A9 cell. **A** Representative immunofluorescence staining images for Piezo1 (green). **B** *Piezo1* mRNA expression was detected by qPCR

Piezo1, a mechanically activated ion channel, plays vital roles in many biological activities [31–37]. It is widely expressed in many cells, including microglia, and can be activated by US [38]. Here, we found that both US and NPs could downregulate *Piezo1* gene expression, consistent with the fact that Piezo1 inhibits neural regeneration [22]. The US-induced *Piezo1* decrease occurs largely because *Piezo1* is sensitive to US. In addition, the MgFe-LDH/NT3 NPs-induced decrease in *Piezo1* expression likely occurs because iron metabolism is closely related to *Piezo1* expression [39], since NPs can release iron ions [19]. To prove our hypothesis, we compared the Piezo1 expression in LPS-induced SIM-A9 treated with MgFe-LDH and MgAl-LDH (Fig. 9A, B). Immunostaining and qPCR results revealed that the Piezo1 expression was significantly increased after LPS stimulation, and no obvious change was observed when treated with MgAl-LDH. However, a significant decrease was found when cells were treated with MgFe-LDH as compared with LPS group, suggesting that iron ions released from MgFe-LDH could downregulate the Piezo1 expression to some extent. Upon the combination of US and NPs, a more notable decrease in *Piezo1* was observed, which was more beneficial for recovery after SCI.

Furthermore, Piezo1 is related to inflammation and neurogenesis, as described in previous studies [22–24]. Piezo1 can also modulate inflammation via the NF-κB pathway [24]; hence, NF-κB was further investigated. We found that the expression of p-NF-κB protein was significantly decreased in the US + NPs group compared to the SCI group, and the expression of related inflammation markers also supported this phenomenon. In conclusion, combined therapy regulates inflammation via the Piezo1/NF-κB pathway.

US as a noninvasive treatment owns great potential for spinal cord nerve repair and cell regeneration. It has been reported that US can promote the proliferation, migration and differentiation of NSCs, and increase the expression of neurotrophic factors and inhibit inflammation. However, US alone is not effective enough, so US is often used in combination with other treatment to exert synergistic therapeutic effects. In this study, our combined therapy shown excellent effects both in vitro and in vivo. It is worth mentioning that the material we used, MgFe-LDH/NT3, is an inorganic 2D material with superior good biocompatibility, its analogue magnesium aluminum carbonate is also used clinically to treat gastric diseases. Thus, combined therapy proposed in our study owns great potential for clinical application.

## Conclusions

**Fig. 10:**
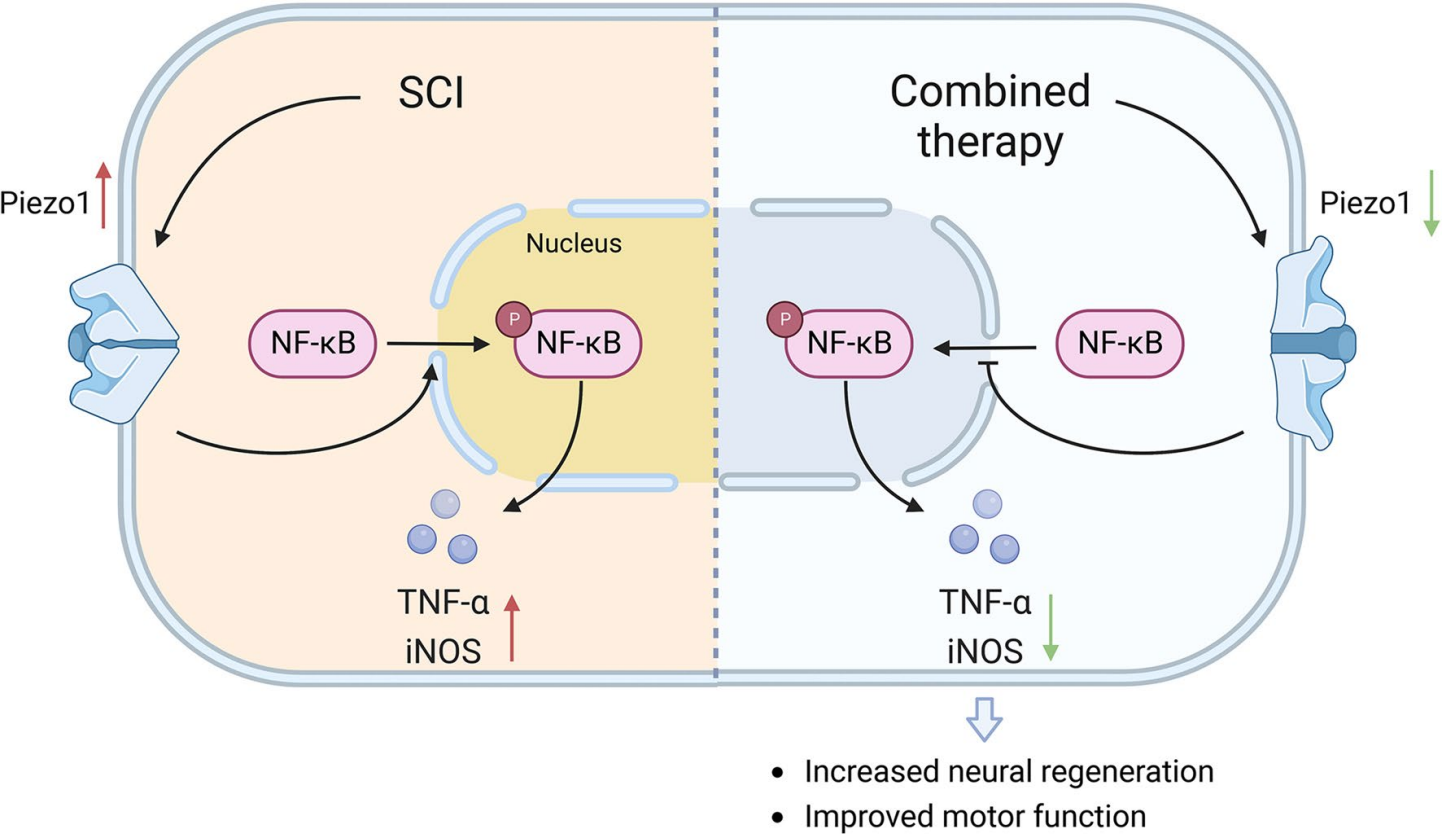
Sequential scheme of the studies performed. MgFe-LDH/NT3 nanoparticles (NPs) were synthesized and then combined with ultrasound (US), and the effects of combined therapy were tested both in vitro and in vivo. In vitro, the roles of promoting neural differentiation and suppressing inflammation were investigated. In vivo, the therapeutic effects of combined therapy on SCI model mice were analyzed, further Piezo1 was identified via RNA-seq analysis and the underlying mechanisms were revealed then. The figure was created with BioRender.com

Here, we implemented a novel strategy combining US and NPs to promote motor function and neurogenesis in mice with SCI and evaluated the treatment’s immunomodulatory function both in vitro and in vivo. To further investigate the potential underlying mechanisms, we performed RNA-Seq to screen for potential DEGs and identified that combined therapy significantly downregulated *Piezo1* expression in the lesion site. Furthermore, we found that the combined therapy mediated *Piezo1* downregulation, which was beneficial for inflammation suppression at the injury site. Altogether, our results suggest that combined therapy results in reduced inflammation and improves motor function better than either US or NPs treatment alone (Fig. 10). This study provides a promising strategy for SCI therapy with potential for future clinical applications.

## Methods

### MgFe-LDH/NT3 synthesis

MgFe-LDH nanomaterials were synthesized based on a previous protocol [40, 41]. Briefly, 1.538 g Mg(NO_3_)_2_·6H_2_O and 0.606 g Fe(NO_3_)_3_·9H_2_O were dissolved in ddH_2_O and poured into a stirring NaOH solution at 60 °C. The suspension was then hydrothermally treated in an autoclave at 100 °C for 16 h. The solution was then centrifuged to precipitate the MgFe-LDH nanomaterials. MgFe-LDH/NT3 was then finally obtained as previously described [10]. Briefly, NT3 was loaded into MgFe-LDH nanomaterials using an ion exchange intercalation method.

### MgFe-LDH/NT3 characterization

The nanomaterial morphology was observed by TEM (JEM-F200, JEOL, Japan) and SEM (Sigma 300, ZEISS, Germany). The phases of MgFe-LDH and MgFe-LDH/NT3 were determined using XRD (X'Pert Pro MPD, Panalytical, Holland). The FTIR spectra of the NPs were recorded by a Bruker FTIR (Nicolet iS20, Thermo Scientific, USA). A Nano Zetasizer (Nano ZS90, Malvern, UK) was used to detect the surface zeta potential and size distribution of the nanomaterials. To detect the concentration of NT3 in supernatant after NT3 was loaded into MgFe-LDH nanomaterials, ELISA Kit for NT3 (bought from cloud clone corp) was used.

### Cell cultures

NSCs were extracted from embryonic Sprague‒Dawley (SD) rats (E14). Telencephalons of each embryo were separated and cut into pieces under a microscope and then incubated with 0.25% trypsin solution (25200,072, Gibco, USA) at 37 °C for 5 min. Then, DMEM/F12 (11330,032, Gibco, USA) containing 10% FBS (044001A, Gibco, USA) was added to neutralize trypsin activity. The cell suspension was centrifuged and resuspended in proliferation medium (DMEM/F12 (11330032, Gibco, USA) containing 2% B27 (17504044, Gibco, USA), 1% N2 (17502001, Gibco, USA), 20 ng/ml bFGF (45033, PeproTech, USA), and 20 ng/mL EGF (31509, PeproTech, USA). Proliferation medium was applied to culture NSCs, and continuous passage culture was performed by incubating with Accutase (07920, STEMCELL, Canada) at 37 °C for 5 min to obtain single cells.

Murine microglial cells (SIM-A9) were purchased from the ATCC (CRL-3265, Manassas, VA, USA). SIM-A9 cells were cultured in DMEM/F12 medium (11330032, Gibco, USA) supplemented with 10% heat-inactivated foetal bovine serum (04-121-1A, BI, Israel) and 5% horse serum (04-004-1A, BI, Israel) under suitable conditions (37 °C, 5% CO_2_). The cells were passaged every three days by incubating with PBS (containing 1 mM EDTA, 1 mM EGTA and 1 mg/mL glucose) for 12 min.

### NSC differentiation

NSCs were seeded into poly-L-ornithine (PLO) (P3655, Sigma, USA)-coated 24-well plates in spontaneous differential medium (DMEM/F12 [11330032, Gibco, USA]) containing 2% B27 (17504044, Gibco, USA), 1% N2 (17502001, Gibco, USA), and 5% FBS [04-001-1ACS, BI, Israel]). To evaluate the differentiation behaviour of NSCs after various treatments, four groups were set: (1) the control group (Ct), which contained NSCs with no further treatment; (2) the US group, which contained NSCs with US treatment only; (3) the MgFe-LDH/NT3 (NPs) group, which contained NSCs with MgFe-LDH/NT3 (NPs) treatment only (1 μg/mL NPs was used); and (4) the US and + MgFe-LDH/NT3 (US + NPs), which contained NSCs treated with US and NPs.

For in vitro cell stimulation experiments, a Cell Ultrasound Stimulator (GV-CSI1.0) provided by GreenValley BrainTech (ShenZhen) Medical Technology Co., Ltd. (China) was used. The US parameters were as follows: frequency = 19.876 MHz, amplitude = 100 mVpp, burst period = 100 ms. According to the manufacturer's instructions, acoustic pressure was measured as 2.45 MPa. NSCs were exposed to US for 5 min per day for three consecutive days. The medium was refreshed every three days. The cells were detected by qPCR and immunofluorescence after seven days of differentiation.

### Anti-inflammation detection in vitro

SIM-A9 cells (1 × 10^5^) were seeded in 24-well culture plates. To study the effects of US and NPs on inhibiting inflammation, five groups were set: (1) For the control (Ct) group, no treatment was given. (2) For the LPS group, SIM-A9 cells were stimulated with 200 ng/mL LPS (L3012, Sigma, USA) 6 h before sample collection. (3) For the LPS + US group, cells were exposed to US, and the duration and parameters of US are described above. (4) For the LPS + NPs group, cells were treated with 1 μg/mL NPs for three days. (5) For the LPS + US + NPs group, cells were treated with US and NPs. The cells were then collected for qPCR and immunofluorescence detection.

### Animals

All animal experiments were approved by and performed in accordance with the standards of the Animal Welfare Committees of Tongji Hospital in Shanghai. Female C57BL/6 mice (~ 20 g, eight weeks old) were obtained from Shanghai JieSiJie Laboratory Animal Company. The experiments were performed after the mice had been allowed to acclimatize to the housing conditions for at least one week.

### Surgical procedure and experimental design

A completely transected SCI model was used in our experiments. The surgical procedure was performed according to a previous report [10]. Briefly, mice were anaesthetized with isoflurane. The T9-10 laminae were exposed at first, and T9-10 laminectomy was performed. Transection was then performed at the T10 level with removal of a 2 mm spinal cord segment. Manual bladder emptying was performed twice daily until autonomic urination.

The animals were then randomized and divided into four groups: (1) the SCI group, which contained SCI mice with no further treatments; (2) NPs group, which contained SCI mice in which NPs were implanted into the lesion site; (3) the US group, which contained SCI mice with US treatment only; and (4) the US + NPs group, which contained SCI mice with US treatment and NPs implantation.

For in vivo mouse US stimulation experiments, the ultrasound neurostimulation system provided by GreenValley BrainTech Medical Technology Co., Ltd. (ShenZhen, China) was used. The mice were anaesthetized with isoflurane and placed in the supine position for US treatment. US coupling gel was applied on the surface of the surgical site. Then, the transducer of the US device was fixed. The US and US + NPs groups were exposed to US 15 min/d for one week. The US was characterized as follows: frequency = 1 MHz, amplitude = 37%, pulse width = 500 μs, pulse repetition period = 7 ms, stimulus duration = 1000 ms, burst period = 5 s. According to the manufacturer's instructions, acoustic pressure was measured as 1.224 MPa.

### Behavioural assessment

The BMS open-field ratings were recorded every week strictly following the double-blind principle for a continuous eight weeks. Bilateral hindlimb movements were determined alone using the BMS rating and averaged.

### Electrophysiological studies

Electrophysiological assays were performed 8 weeks after the operation as described previously [29]. MEPs can comprehensively reflect the locomotor function of an animal and indicate neurological recovery in SCI.

### RNA sequencing

RNA was extracted according to a previously published report [40] and then sent to BGI Tech for quality control and RNA sequencing on their platform. The results were then analysed using a BGI Dr. Tom online system according to a standard protocol. DEGs between the SCI and US + NPs groups were chosen for further analysis and subjected to Kyoto Encyclopedia of Genes and Genomes (KEGG) pathway analysis, Gene Ontology (GO) enrichment, and GSEA for gene annotation and vital gene or pathway screening.

### Immunofluorescence staining

Immunofluorescence staining of cell samples was performed as follows: Cells (NSCs or SIM-A9) were fixed with 4% PFA, permeabilized with 0.3% Triton X-100, blocked in 5% donkey serum and incubated with the primary antibody at 4 °C overnight. After fluorescent secondary antibody incubation and DAPI staining, the cells were micrographed by a Leica confocal microscope (LSM 700, Carl Zeiss, Jena, Germany).

For immunofluorescence of tissue samples, the procedure was as follows: isoflurane was applied as anaesthesia to the mice, and the mice were intracardially perfused with PBS and then fixed with 4% paraformaldehyde. The spinal cord (~ 1.5 cm) within the injury site was dissected, fixed with 4% paraformaldehyde, and dehydrated with 30% sucrose solution. Coronal Sects. (10 μm thick) were cut using a cryostat (CM3050S, Leica, Germany). Sections were incubated for 10 min with 0.25% Triton X-100 to permeabilize the samples. Then, the sections were incubated with primary antibodies at 4 °C overnight after blocking. After fluorescent secondary antibody incubation and DAPI staining, the cells were micrographed by confocal microscopy (LSM 700, Carl Zeiss, Jena, Germany).

The antibodies used were as follows: TNF-α (1:500, polyclonal, rabbit, Bioss, Cat# bs-10802R), NeuN (1:500, Guinea pig, polyclonal, Millipore, Cat# ABN90P, RRID: AB_2341095), MAP2 (1:500, polyclonal, rabbit, Abcam, Cat# ab32454, RRID: AB_776174), Piezo1 (1:200, rabbit, Novus, Cat# NBP1-78537, RRID: AB_11003149), and β3-tubulin (1:500, monoclonal, rabbit, Cell Signaling Technology, Cat#5568S) antibodies.

The secondary antibodies used were as follows: polyclonal secondary donkey anti-mouse Alexa Fluor^™^ Plus 488 (Thermo Fisher Scientific, Cat# A32766, RRID: AB_2762823), polyclonal secondary donkey anti-rabbit Alexa Fluor^™^ Plus 488 (Thermo Fisher Scientific, Cat# A32790, RRID: AB_2762833), and Alexa Fluor 594-AffiniPure Donkey Anti-Guinea Pig IgG (Jackson ImmunoResearch Labs, 706585148, Catalogue No. NC0452490).

### mRNA extraction and RT‒qPCR analysis

RNA was extracted from samples with RNAiso Plus (9108, Takara, Japan) reagent according to the manufacturer’s instructions. RNA concentration was detected via a NanoDrop (Thermo Fisher Scientific, USA). Reverse transcription was performed using the Primer Script Reverse Transcriptase Kit (RR037A, Takara, Japan). Quantitative real-time PCR (qPCR) was performed using SYBR Premix (RR420A, Takara, Japan) on a QuantStudio 3 Real-Time PCR instrument (Thermo Fisher Scientific, USA).

### Western blot

Cells or tissues were collected and lysed using RIPA lysis buffer (P0013B, Beyotime, China) supplemented with protease and phosphatase inhibitors (P1050, Beyotime, China) according to the manufacturer’s instructions. After protein quantification using a BCA Protein Assay Kit (KeyGEN), 20 μg of protein lysates was loaded, separated by electrophoresis, transferred onto a polyvinylidene difluoride (PVDF) membrane (IPVH00010, Merck Millipore, USA), blocked with 5% BSA for 1 h, and incubated with specific primary antibodies overnight at 4 °C. After incubation with secondary antibodies for 1 h at room temperature, the membranes were developed with chemiluminescence. A chemiluminescence kit (P90720, Millipore, USA) was applied, and the bands were observed with a chemiluminescence detection system (Tanon-5200Multi, Tanon, Shanghai, China).

The primary antibodies used were as follows: β-Actin (1:5000, mouse anti-mouse, Proteintech, Cat# 66009-1-Ig, RRID: AB_2687938), NF-KappaB p65, phospho (Ser536) (1:1000, rabbit anti-mouse, Cell Signaling Technology, Cat# 3033, RRID: AB_331284) and NF-κB p65/RelA (1:1000, rabbit anti-mouse, Cell Signaling Technology, Cat# 8242, RRID: AB_10859369) antibodies. The secondary antibodies used were as follows: a goat anti-mouse IgG secondary antibody (1:3000, Abcam Cat# ab6789, RRID:AB_955439) and a goat anti-rabbit IgG secondary antibody (1:3000, Abcam Cat# ab6721, RRID: AB_955447).

### Haematoxylin and eosin (H&E) staining

Standard H&E staining of visceral tissue was performed. Briefly, tissues were immersed in paraffin first, and then paraffin-immersed samples were sliced into 10 µm-thick sections with a microtome (RM2235, Leica, Germany). The paraffin sections were stained following standard procedures. Images were acquired on a microscope (BX53, Olympus, Japan).

### Cell counting kit-8 (CCK-8) assay

CCK-8 was applied to detect the cell viability of NSCs and SIM-A9 with different treatment according to the manufacturer's instructions. First, the cells were seeded into 24-well plates overnight. Wells with no cells but medium only were set as the blank control. Subsequently, cells were exposed to different treatment. 24, 48 or 72 h later, cells were incubated with 50 µL CCK-8 solution for additional 3 h at 37 °C. The absorbance was recorded at 450 nm by a microplate reader (Thermo Fisher Scientific Inc.), and the cell viability in each group was calculated accordingly, as compared with the control group (100%).

### Statistical analysis and figure preparation

All results are presented as the mean ± standard deviation (SD), and the statistical significance for each experiment was determined using one-way analysis of variance (ANOVA). *, **, ***, and **** represent p < 0.05, p < 0.01, p < 0.001, and p < 0.0001, respectively.

## Data Availability

All data generated or analyzed during this study are included in this published article.
